# Vacuum phenomenon as a predictor of kyphosis after implant removal following posterior pedicle screw fixation without fusion for thoracolumbar burst fracture: a single-center retrospective study

**DOI:** 10.1186/s12891-022-05051-z

**Published:** 2022-01-27

**Authors:** Masahiro Hirahata, Tomoaki Kitagawa, Youichi Yasui, Hiroyuki Oka, Iwao Yamamoto, Kazuaki Yamada, Muneyoshi Fujita, Hirotaka Kawano, Keisuke Ishii

**Affiliations:** 1 Department of Orthopaedic Surgery, Teikyo University School of Medicine, 2-11-1 Kaga, Itabashi-ku, Tokyo, 1738605 Japan; 2 Department of Medical Research and Management for Musculoskeletal Pain, 22nd Century Medical and Research Center, The University of Tokyo, Tokyo, Japan; 3 Trauma and Reconstruction Center, Teikyo University Hospital, Tokyo, Japan

**Keywords:** Implant removal, Kyphotic deformity, Posterior pedicle screw fixation, Thoracolumbar burst fracture, Vacuum phenomenon

## Abstract

**Background:**

Posterior pedicle screw fixation without fusion has been commonly applied for thoracolumbar burst fracture. Implant removal is performed secondarily after bone union. However, the occurrence of secondary kyphosis has recently attracted attention. Secondary kyphosis results in poor clinical outcomes. The purpose of this was to determine predictors of kyphosis after implant removal following posterior pedicle screw fixation without fusion for thoracolumbar burst fracture.

**Methods:**

This retrospective study reviewed 59 consecutive patients with thoracolumbar burst fracture who underwent implant removal following posterior pedicle screw fixation without fusion. Inclusion criteria were non-osteoporotic fracture and T11-L3 burst fracture. Old age, sex, initial severe wedge deformity, initial severe kyphosis, and vacuum phenomenon were examined as factors potentially associated with final kyphotic deformity (defined as kyphotic angle greater than 25°) or loss of correction. Logistic regression analysis was performed using propensity score matching.

**Results:**

Among the 31 female and 28 male patients (mean age 38 years), final kyphotic deformity was found in 17 cases (29%). Multivariate analysis showed a significant association with the vacuum phenomenon. Loss of correction was found in 35 cases (59%) and showed a significant association with the vacuum phenomenon. There were no significant associations with other factors.

**Conclusions:**

The findings of this study suggest that the vacuum phenomenon before implant removal may be a predictor of secondary kyphosis of greater than 25° after implant removal following posterior pedicle screw fixation without fusion for thoracolumbar burst fracture, but that old age, sex, initial severe kyphosis, and initial severe wedge deformity may not be predictors.

## Background

Thoracolumbar burst fractures are defined as injuries of the anterior and middle spinal columns [1]. In terms of incidence, they account for 30–64% of all thoracolumbar fractures [2, 3]. The optimal treatment strategy for these injuries remains controversial, beginning with choosing between surgical or non-surgical treatment methods, although the recent trend is to move away from non-surgical treatment [4, 5].

Among the various surgical techniques reported for thoracolumbar burst fracture, posterior pedicle screw fixation without fusion has been commonly applied [6] and its effectiveness has been reported [7]. A recent meta-analysis suggested there is no significant difference between non-fusion and fusion in terms of radiological outcome, functional outcome, neurologic improvement, or implant failure rate, but non-fusion is associated with significantly reduced operative time and blood loss [8]. Also, implant removal, which is performed secondarily after bone union, decreases the stiffness of the fixed segment, which in turn could alleviate the concentration of stress in adjacent segments [9, 10]. However, the occurrence of secondary kyphosis has recently attracted attention, with reports that 29–43% of patients developed it after implant removal [11, 12]. Secondary kyphosis results in poor clinical outcomes, and yet little information is available about the predictors of kyphosis in these patients following implant removal after bone union [13].

The aim of this study was to determine predictors of kyphosis after implant removal following posterior pedicle screw fixation without fusion for thoracolumbar burst fracture.

## Methods

This retrospective study was approved by the institutional review board and was performed in strict accordance with the ethical standards stipulated in the 1964 Declaration of Helsinki and its later amendments. All subjects had provided written informed consent for their data to be used for research purposes. A chart review was conducted of 59 consecutive patients with thoracolumbar burst fracture who underwent implant removal following posterior pedicle screw fixation without fusion at our trauma center between December 2008 and June 2016. None of the patients underwent anterior fixation. A single surgeon performed all surgical procedures and decided the preoperative and postoperative care. Surgery for implant removal was indicated based on radiological union of the fractured vertebral body identified on computed tomography (CT) and more than 10 months of follow-up after posterior fixation. Implant removal was contraindicated in patients with altered consciousness. Inclusion criteria were non-osteoporotic fracture and T11-L3 burst fracture. Exclusion criteria were two or more affected vertebral bodies and fixation five or more segments.

### Surgical technique

#### Posterior pedicle screw fixation without fusion

After induction of general anesthesia, the patient was placed in a prone position supported on a spinal surgery Table. A midline incision was placed to allow thorough exposure of the posterior elements. Pedicle screws were placed using standard landmarks and techniques. Bilateral rods were pre-contoured and positioned into the pedicle screws. The wound was then irrigated and closed. Postoperatively, all patients were allowed to sit up as soon as they were fitted with a custom-molded thoracolumbar brace. The brace was used for 3 months after the surgery.

#### Implant removal

All patients underwent a standard surgical procedure. After induction of general anesthesia, the patient was placed in a prone position supported on a spinal surgery Table. A midline incision of the same length as that used for the posterior fixation was placed. Pedicle screws and bilateral rods were removed. Postoperatively, all patients were allowed to stand as soon as they had been fitted with a custom-molded thoracolumbar brace. The brace was worn for 3 months after implant removal.

### Radiographic assessment

**Fig. 1:**
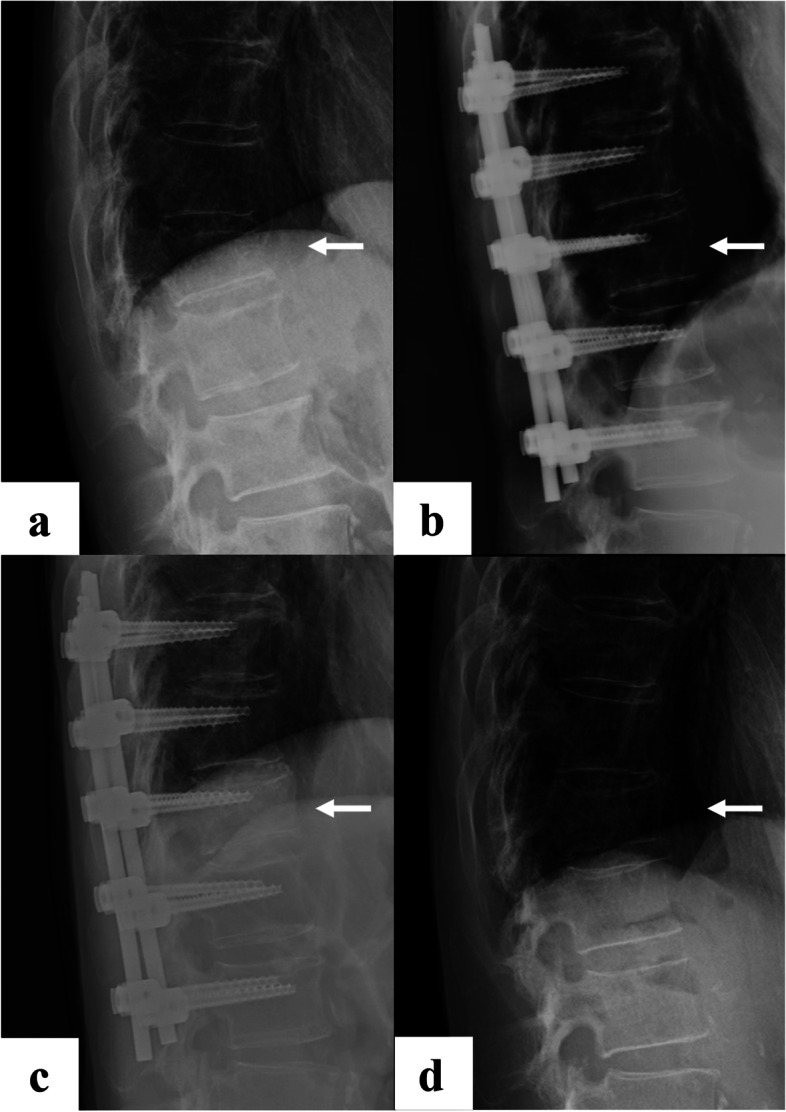
**a**: at the time of injury, **b**: after posterior pedicle screw fixation, **c**: before implant removal, and d: at final observation

**Fig. 2:**
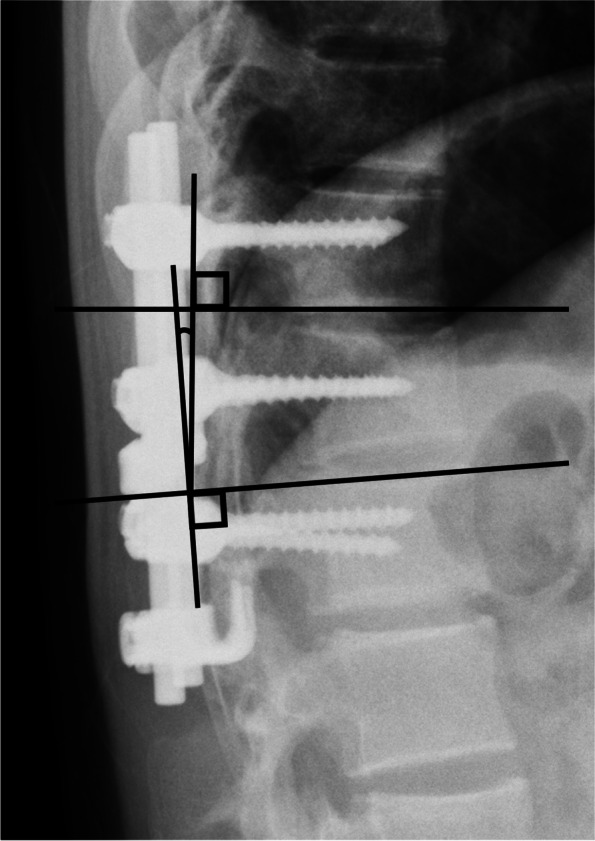
Kyphotic angle

**Fig. 3:**
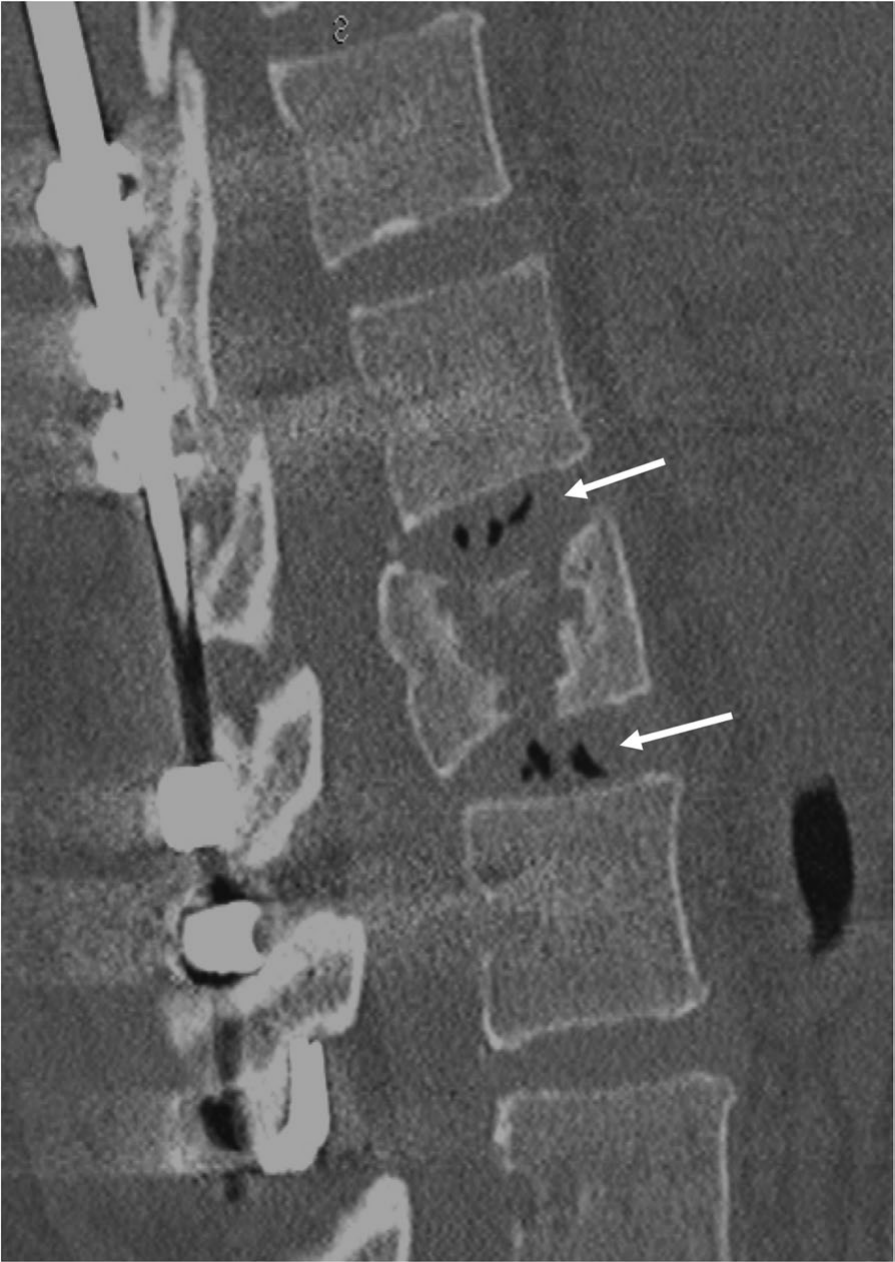
Vacuum phenomenon

Radiographic assessment was performed using lateral thoracolumbar spine radiographs taken at four timepoints: at the time of injury, after posterior pedicle screw fixation, before implant removal, and at final observation (Fig. 1). Kyphotic angle was defined as the Cobb angle of the lower endplate line of the cranial vertebral body and the upper endplate line of the caudal vertebral body based on the fractured vertebral body (Fig. 2). The vacuum phenomenon was evaluated on sagittal CT images before implant removal (Fig. 3). All measurements were made by a board-certified orthopedic surgeon who was not involved in the patients’ care.

### Evaluation of predictors of kyphosis

Logistic regression analysis was performed to determine predictors of kyphosis following implant removal in our series. The objective variable was final kyphotic deformity or loss of correction. The explanatory variables were old age, sex, initial severe kyphosis, initial severe wedge deformity, and the vacuum phenomenon.

**Fig. 4:**
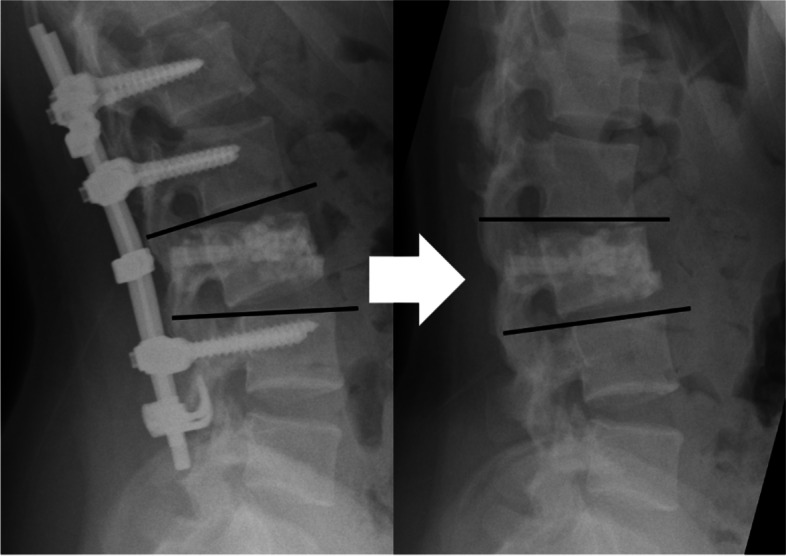
Loss of correction

Final kyphotic deformity was defined as a kyphotic angle greater than 25° at the final observation, based on a previous study in which patients with kyphosis greater than 25° had poor clinical outcomes [13]. Loss of correction was defined as an increase in the kyphotic angle greater than 15° at the final observation compared with kyphotic angle after posterior fixation (Fig. 4).

Old age, sex, initial severe wedge deformity, initial severe kyphosis, and the vacuum phenomenon were examined as factors associated with kyphotic deformity or loss of correction. Old age was defined as 60 years or older at the final observation. Initial severe wedge deformity was defined as a wedge angle of the fractured vertebral body greater than 25° at the time of injury. Initial severe kyphosis was defined as a kyphotic angle greater than 25° at the time of injury. The vacuum phenomenon was defined as the radiographic finding of an air-density linear radiolucency in the intervertebral disc adjacent to the fractured vertebral body before implant removal [14]. Tash et al. suggested that the vacuum phenomenon was caused by herniation of disc material and was then responsible for vaporization in vertebral fractures [15]. AO fracture classification, especially type C, was not examined as a factor because Chen et al. reported that fracture type was not a risk factor of kyphosis recurrence after implant removal in thoracolumbar burst fractures following posterior fixation [16]. Vertebroplasty and laminectomy were not also examined as a factor. Aono et al. reported that kyphosis recurred due to adjacent discs injury by the original trauma, with or without vertebroplasty [17].

### Statistical analysis

Statistical analysis was performed with SPSS statistical software (version 21.0; IBM Corp., Armonk, NY). *P* values below 0.05 were considered to indicate significance. Logistic regression analysis was performed with old age, sex, initial severe kyphosis, initial severe wedge deformity, and vacuum phenomenon as the explanatory variables and final kyphotic deformity as the objective variable. Because of possible differences between those patients with final kyphotic deformity compared with those without it, we chose to mitigate such confounding by using propensity score matching. The matched variables included old age, sex, initial severe kyphosis, initial severe wedge deformity, and the vacuum phenomenon. The same analysis was performed with these factors as explanatory variables and loss of correction as the objective variable.

## Results

**Fig. 5:**
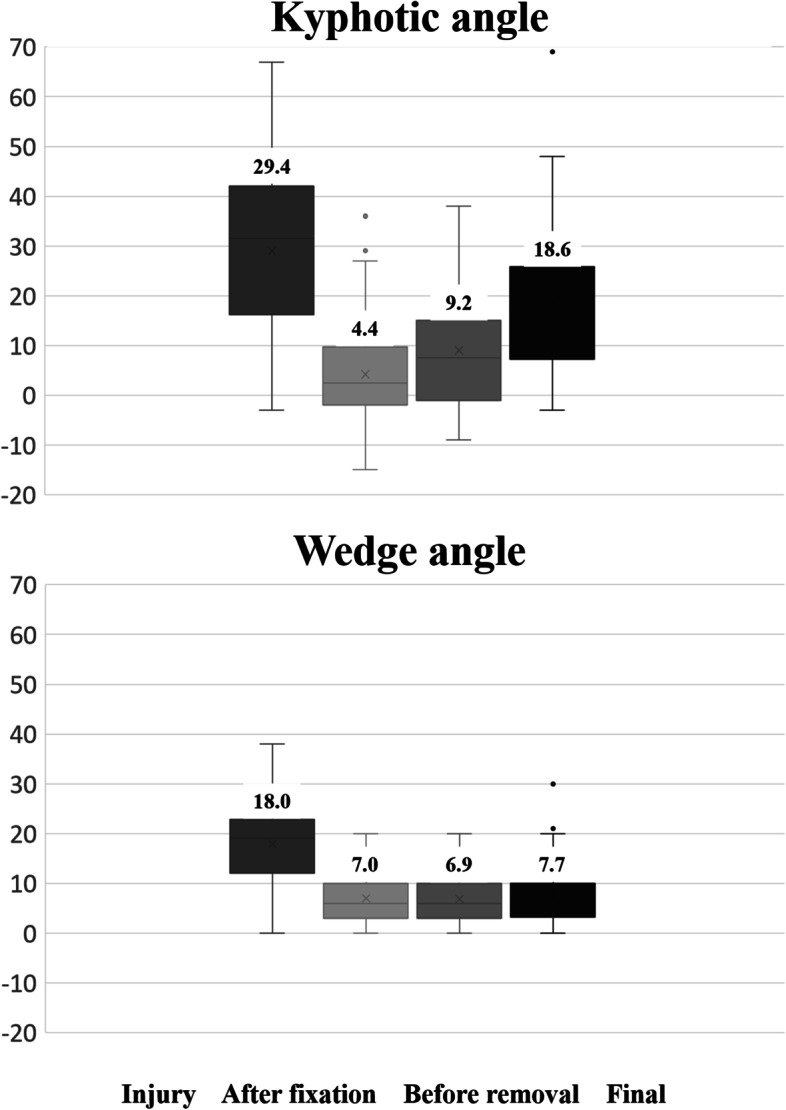
The mean of kyphotic and wedge angle at four timepoints

Table 1 shows patient demographics and clinical characteristics for the 31 female and 28 male patients (mean age 38 [range, 17–68] years) reviewed in this study. Mean time from the time of injury to the final observation was 32 months, that from the time of injury to implant removal was 16 months, and that from implant removal to the final observation was 15 months (Table 2). Radiographic assessment revealed that implant removal did not significantly affect initial severe wedge deformity but did significantly affect the kyphotic angle (Fig. 5). Thus, the kyphotic angle after implant removal was affected by the intervertebral disc.

**Table 1:** Analyzed variables with percentages

	All (*n* = 59)	Percentage
Age	38 (17–68)	
Male sex	30	50.8
Level		
T 11 / 12	4 / 8	6.8 / 13.6
L 1 / 2 / 3	26 / 14 / 7	44.1 / 23.7 / 11.9
Classification		
A 1 / 2 / 3	4 / 4 / 26	6.8 / 6.8 / 44.1
B 1 / 2 / 3	3 / 11 / 1	5.1 / 18.6 / 2.7
C 1 / 2 / 3	6 / 2 / 2	10.2 / 3.4 / 3.4
Initial severe kyphosis	9	15.0
Wedge deformity	13	21.7
Instrumentation		
1 above - 1 below	10	16.7
2 above - 1 below	42	70.0
2 above - 2 below	7	11.9
Vertebroplasty	35	59.3
Laminectomy	6	10.2
Vacuum phenomenon	29	49.2

**Table 2:** Average intervals with ranges

	Average	Range
Injury - Removal	16	10–41
Removal - Final	15	1–41
Injury - Final	32	12–74

Final kyphotic deformity was found in 17 cases (29%). Multivariate analysis showed a significant positive association with the vacuum phenomenon (odds ratio [OR]: 7.2, 95% confidence interval [CI]: 1.5–34.7, *P* = 0.01). Multivariate analysis showed no associations with old age, sex, initial severe kyphosis, or initial severe wedge deformity (Table 3). Loss of correction was found in 35 cases (59%), and multivariate analysis showed a significant positive association with the vacuum phenomenon only (OR: 3.9, 95% CI: 1.2–13.1, *P* = 0.03; Table 4).

**Table 3:** Final kyphotic deformity

	Final kyphotic deformity		OR	95% CI	*P* value
	(+); *n* = 17	(−); *n* = 42			
Old age	3	2	8.6	0.9–85.2	0.07
Female	5	26	0.3	0.1–1.1	0.06
Initial severe kyphosis	10	29	0.6	0.1–3.0	0.56
Severe wedge deformity	5	8	4.0	0.7–21.6	0.11
Vacuum phenomenon	11	18	7.2	1.5–34.7	0.01

*OR* odds ratio, *CI* confidence interval

**Table 4:** Loss of correction

	Loss of correction		OR	95% CI	*P* value
	(+); *n* = 35	(−); *n* = 25			
Old age	3	2	3.8	0.5–30.6	0.21
Female	10	21	0.5	0.2–1.7	0.27
Initial severe kyphosis	16	23	1.7	0.5–6.0	0.45
Severe wedge deformity	5	8	0.9	0.2–3.8	0.90
Vacuum phenomenon	15	14	3.9	1.2–13.1	0.03

*OR* odds ratio, *CI* confidence interval

## Discussion

This study found that the vacuum phenomenon of the intervertebral disc adjacent to the fractured vertebral body before removal of the posterior pedicle screw was associated with secondary severe kyphotic deformity in patients after implant removal for thoracolumbar burst fracture. No such association was seen with old age, sex, the initial severe kyphosis, or initial severe wedge deformity. These findings should be helpful in the clinical setting because there has been little consensus to date on what constitutes routine implant removal in the context of healed fracture. Even though implant removal is probably common practice among spine surgeons after bone union, because it decreases the stiffness of the fixed segment and reduces the concentration of stress in the adjacent segments [16], the recurrence of kyphotic deformity is a recognized shortcoming of implant removal following posterior fixation, affecting 29–43% of patients [9, 10]. Given that the prognostic factors for secondary kyphotic deformity have not been established yet, our findings can serve as a reference to assist physicians in deciding whether implant removal should be conducted or not.

In this study, final kyphotic deformity and loss of correction were set as the objective variables. To our knowledge, only two previous studies have investigated the predictors of kyphosis following implant removal in posterior pedicle screw fixation without fusion for thoracolumbar burst fracture [17, 18]. These studies set loss of correction alone as the objective variable, although the clinical significance of loss of correction following implant removal is as yet unclear in the literature [17, 18]. In contrast, final kyphotic deformity has previously been proposed to affect clinical outcomes. Kraemer et al. evaluated functional outcome in 24 patients with a minimum of 2 years of follow-up after thoracolumbar burst fracture without neurologic deficit using the SF-36 survey and the Roland scale and concluded that clinical outcomes in patients with kyphosis greater than 25° were poor [13].

In relation to loss of correction, this study demonstrated that initial severe kyphosis was not associated with such loss. Additionally, the kyphotic angle after implant removal was presumed to be affected by the intervertebral disc. Previous studies have reported varying outcomes for initial severe kyphosis. The reason for the lack of significant association between initial severe kyphosis and loss of correction might be because initial severe kyphosis was mainly affected by the initial severe wedge deformity of the fractured vertebral body. In a retrospective comparative study, Chen et al. did find an association between the two. In their study, they defined loss of correction as an increase of more than 5° in the kyphotic angle at the final observation compared with the kyphotic angle after posterior fixation. Initial severe kyphosis was evaluated using the percentage of the anterior vertebral body height with respect to the posterior vertebral body height of the fractured vertebra and the percentage of the anterior vertebral body height with respect to mean anterior height of the upper and lower adjacent vertebrae [17]. Also, in a comparative retrospective study by Aono et al., initial severe kyphosis at the time of injury was associated with loss of correction. In their study, they used an increase of more than than 10° in the kyphotic angle at the final observation compared with kyphotic angle after posterior fixation, and they evaluated initial severe kyphosis in the same way as in our study [18]. We decided to use an increase of more than 15° in our study based on a review article by Mazel et al. in which thoracolumbar burst fracture with kyphosis of 15° or more was considered to require correction [19].

This study revealed an association of the vacuum phenomenon of the intervertebral disc adjacent to the fractured vertebral body before removal of the posterior pedicle screw with secondary severe kyphotic deformity following implant removal for thoracolumbar burst fracture. The vacuum phenomenon may be the result of disc degeneration. Previous studies have shown that initial severe kyphosis resulted from loss of not only vertebral height but also disc height and that initial vertebral height at the time of injury was corrected after primary surgery and maintained after implant removal [20, 21]. Similarly, the fractured vertebral angle was maintained after implant removal in this study. On the other hand, the angle of the intervertebral disc adjacent to the fractured vertebral body at the time of injury increased after posterior fixation but was not maintained after implant removal. This loss of disc angle may be due to degeneration of the disc, which might be accelerated by traumatic damage at the time of injury. Similarly, Kanezaki et al. reported that the association of severe disc damage at the time of injury, as evaluated with magnetic resonance imaging (MRI), with kyphosis after implant removal [22]. While the outcome from their study is valuable, CT imaging is more convenient in daily practice than MRI. At our institution, MRI evaluation is indicated for patients with neurological deficit, not for all patients with thoracolumbar burst fracture. In our institution, the CT was taken at the time of injury and before removing the implant. However, we used the CT before implant removal in the present study because none had the vacuum phenomenon at the time of injury. We believe our CT findings in this study can prove valuable for the many physicians who use CT in daily practice and the patients who do not receive an evaluation by CT or MRI at the time of injury.

Old age, which was defined as age above 60 years, was not associated with kyphotic deformity in this study, in contrast with the results from Chen et al. In their study, patients were divided into recurrence and non-recurrence groups based on loss of correction. Mean age was significantly higher in the recurrence group (40.1 ± 9.5 years) than in the non-recurrence group (35.3 ± 8.1 years) [17]. They discussed that loss of correction was associated with patient age due to age-related loss of bone density. The definition of old age in the present study was more reflective of loss of bone density than in their study and age was not associated with kyphotic deformity in this study. The difference between these studies suggests caution is needed in interpreting the effect of age on secondary kyphotic deformity following implant removal in posterior pedicle screw fixation.

This study provides new criteria for physicians to decide whether or not to perform implant removal. Because final kyphotic deformity is suggested to cause poor clinical outcomes, physicians can cautiously consider the advantages and disadvantages of removing the pedicle screw for patients with the vacuum phenomenon apparent on CT examination [13]. Several studies have reported on stem cell-induced regeneration of the intervertebral disc [23], and further studies may be needed to better understand the potential effect of regenerative treatment.

The present study has several limitations. First, the data was obtained retrospectively. Second, functional outcomes were not evaluated. Third, patients were selected from a single institution. Fourth, many fracture types and fixation methods were included in the present study. In addition to these, there were various other biases, such as vertebroplasty and laminectomy. Lastly, the study design was not a comparative study, and it should be compared to the non-removal group in the future.

## Conclusion

The findings of this study suggest that the vacuum phenomenon before implant removal may be a predictor of secondary kyphosis of greater than 25° after implant removal following posterior pedicle screw fixation without fusion for thoracolumbar burst fracture, but that old age, sex, initial severe kyphosis, and initial severe wedge deformity may not be predictors. Due to the nature of the study design, future study, such as a multicenter prospective randomized study, is warranted to provide a more robust finding of risk factors for kyphosis deformity.

## Data Availability

The datasets generated during and analyzed during the current study are not publicly available due to disagreement of participants for their data to be shared but are available from the corresponding author on reasonable request.

## Abbreviations

CT: Computed tomography
OR: Odds ratio
CI: Confidence interval
MRI: Magnetic resonance imaging
